# Protective Effect of Ethanol Extract of *Gymnosporia montana* (Roth) Bemth. in Paracetamol-induced Hepatotoxicity in Rats

**DOI:** 10.4103/0250-474X.70493

**Published:** 2010

**Authors:** Parvati B. Patel, T. K. Patel, P. Shah, Seema N. Baxi, H. O. Sharma, C. B. Tripathi

**Affiliations:** Department of Pharmacology, Government Medical College, Bhavnagar - 364 001, In3dia; 1 Department of Biochemistry, Government Medical College, Bhavnagar - 364 001, India; 2 Department of Pathology, Government Medical College, Bhavnagar - 364 001, India

**Keywords:** Ethanol extract, *Gymnosporia montana* (Roth) Bemth., hepatoprotective activity, paracetamol

## Abstract

The aim of the present study was to explore the hepatoprotective activity of the ethanol extract of leaves of *Gymnosporia montana* (Roth) Bemth. (Family: Celastraceous) against paracetamol-induced hepatotoxicity. Hepatotoxicity in Wistar rats was induced by a single intraperitoneal dose of 500 mg/kg of paracetamol and studied by comparing parameters such as serum glutamate oxaloacetate transaminase, serum glutamate pyruvate transaminase, alkaline phosphatase and histopathological examination of liver. Pre and post-treatment with ethanol extract of *Gymnosporia montana* (Roth) Bemth. at doses of 50 and 100 mg/kg was studied by comparing the above mentioned parameters with silymarin (100 mg/kg) as standard. Both doses of ethanol extract of *Gymnosporia montana* (Roth) Bemth. were found to be hepatoprotective. Extract at the dose of 100 mg/kg produced effects comparable to those of silymarin. The present study indicates that alcohol extract of *Gymnosporia montana* (Roth) Bemth. possessed significant hepatoprotective activity.

The liver is an organ of paramount importance, which plays an essential role in the metabolism of foreign compounds entering the body and fight against disease, supply nutrient and energy[1]. The liver is expected not only to perform physiological functions but also to protect against the hazards of harmful drugs and chemicals[2,3]. The liver function affects almost every organ system in the body and liver malfunction causes serious health problems in human being. Most common causes of liver diseases are viral infection[4,5] and drugs[6,7].

The drugs available in modern system of medicine provide only symptomatic relief. In the absence of reliable hepatoprotective drugs in allopathic medical practices, there are number of herbal drugs and their formulations that have been claimed to have curative effects and play a role in the management of various liver disorders in ethno medical practices and in traditional system of medicine (Ayurveda) in India[8–10]. However, we do not have satisfactory remedies for serious liver diseases. So the search for effective hepatoprotective drug continues.

*Gymnosporia montana* (Roth) Bemth. (GM) is a traditional herbaceous plant of Celastraceous family found in different regions of India[11]. In vernacular language it is called as *Vikalo*. It is used to purify the blood; cures peptic ulcer and haemorrhoids. It relieves *Kapha*, inflammation, burning sensation, thirst and corneal opacity. It is also used in treatment of snake bite and pediculosis[12]. The pulverized leaves are given in milk to children as vermifuge and decoction of the leafy twig is used in mouth wash to relieve toothache. Paracetamol-induced hepatoxicity in rats has been used in several investigations[13,14]. The present study was carried out to evaluate the hepatoprotective activity of *Gymnosporia montana* (Roth) Bemth. extract in paracetamol-induced (PCM) hepatotoxicity in Wistar rats and compared with standard drug silymarin.

The research project was started after obtaining clearance from Institutional Animal Ethics Committee, Government Medical College, Bhavnagar (Gujarat), India. (CPCSEA registration no. is 485/01/c/CPCSEA, Dated: 31^st^ October, 2001). Wistar rats of either sex weighing 150-250 g were procured from central animal house of the institute. They were housed in clean polypropylene cages under standard conditions (temperature-controlled room: 24±2°; RH: 60-70%) with 12 h light-dark cycles and given standard pellet diet and water *ad libitum*. Food was withdrawn 12 h before the experiments.

Parenteral preparation of paracetamol (Inj. Febrinil, Svizera Health Care, Mumbai, India), silymarin (Sigma) and serum glutamate oxaloacetate transaminase (SGOT), serum glutamate pyruvate transaminase (SGPT), alkaline phosphates (ALP, Transgenic Company, Ahmedabad, India) measurement kits were used for the study. The leaves of *Gymnosporia montana* (Roth) Bemth. were collected from Victoriya Park an urban forest of District Bhavnagar, Gujarat. It was identified and authenticated at the Department of Botany, Bhavnagar University, Bhavnagar, Gujarat. The leaves of *Gymnosporia montana* (Roth) Bemth. were plucked, air-dried in shade, powdered and stored in air-tight containers. The powder was extracted with 95% ethanol in Soxhlet apparatus. The extract was concentrated under vacuum to get the residue. The residue was dried in desiccator containing silica gel and stored in refrigerator at 4°. The *Gymnosporia montana* (Roth) Bemth. (25 mg/ml) and silymarin (25 mg/ml) suspensions were freshly prepared on each day in 10% ethanol and double distilled water, respectively. Paracetamol ampoules (150 mg/ml) were used as such.

Rats were divided into nine groups with six rats in each group. Group I served as a control group, received vehicle (10% ethanol p.o.) for 7 d. In post-treatment groups, II to V, all the animals received a single dose of paracetamol 500 mg/kg intraperitonealy (i.p.) followed by treatment with either vehicle (10% ethanol p.o.) or silymarin (100 mg/kg p.o.) or 2 doses (50 and 100 mg/kg p.o.) of *Gymnosporia montana* (Roth) Bemth. o.d. for 7 d.

In pre-treatment groups, VI to IX, all the animals were treated with vehicle (10% ethanol p.o.) or silymarin (100 mg/kg p.o.) or 2 doses of (50 and 100 mg/kg p.o.) of *Gymnosporia montana* (Roth) Bemth. once daily for 7 d according to their group followed by a single dose of paracetamol 500 mg/kg i.p. one h after the last dose of study drugs.

Blood samples were collected from each animal from the intra orbital plexus with the help of thin glass capillary under pentobarbitone sodium (30 mg/kg i.p.) anesthesia after 24 h of the last dose of treatment. Serum was separated for estimation of biochemical parameters, SGOT, SGPT and ALP by UV kinetic test-optimized International Federation of Clinical Chemistry (IFCC) method in fully automated analyzer[15].

The animals were sacrificed soon after blood collection, cut open to remove the liver, perfused and then stored in 10% neutral formalin for 24 h. Then 5 mm thick pieces of the liver were embedded in paraffin, cut into 5 μm thick sections with microtome, stained using haematoxylin-eosin dye and finally mounted in dibutyl diesterate parathylate xylene. Sections were observed under microscope for histological changes in liver architecture and their photomicrographs were taken.

The results obtained for biochemical parameters were expressed as mean±SEM. Unpaired t test was performed between control and toxin control groups. One-way ANOVA and Dunnett’s Multiple Comparison Test was performed using GraphPad Instat software demo version between toxin control group and test groups. *p*<0.05 was considered significant.

The results are presented in Table 1 and 2. The level of marker enzymes, SGOT, SGPT and ALP were significantly increased in pre- and post-treated paracetamol groups as compared to normal control group (*p*<0.05). The groups, pre and post-treated with *Gymnosporia montana* (Roth) Bemth., at doses of 50 and 100 mg/kg showed significant reductions in the levels of serum marker enzymes as compared to the paracetamol control group (*P*<0.05).

**TABLE 1:** EFFECT OF POST-TREATMENT OF ETHANOL EXTRACT OF *GYMNOSPORIA MONTANA* IN PARACETAMOL-INDUCED HEPATOTOXICITY IN RATS

Groups	SGOT (u/I)	SGPT (u/I)	ALP (u/I)
Control	100 ±5.6	52.7±3.6	182.8± 5.9
Toxin control (PCM + vehicle)	327.1±25.3@	184.2±9.5@	781.8±118.6@
PCM + silymarin (100 mg/kg)	264.5±17.1*	105.3±11.2*	517.0±18.4*
PCM + GM (50 mg/kg)	210.2±6.3*	116.7±4.8*	642.3±48.9
PCM + GM (100 mg/kg)	263.0±12.4*	111.3±13.1*	514.8±60.9*
One-way ANOVA F (df)	8.2 (3,20)	12.7 (3,20)	3.13 (3,20)

Value are Mean±SEM; n= 6

@ *p*<0.05, When compared to control vs toxin control group

* *p*<0.05, when compared to toxin control

**TABLE 2:** EFFECT OF PRE-TREATMENT OF ETHANOL EXTRACT OF *GYMNOSPORIA MONTANA* IN PARACETAMOL-INDUCED HEPATOTOXICITY IN RATS

Groups	SGOT (u/I)	SGPT (u/I)	ALP (u/I)
Control	100 ±5.6	52.7±3.6	182.8± 5.9
Toxin control (vehicle +PCM)	388.7±40.9@	166.2±24.3@	580.5±145.8@
Silymarin(100 mg/kg) + PCM	232.17±9.7*	93.8±6.0*	341.2±27.8
GM (50 mg/kg) + PCM	277.0±11.7*	199.7±11.5	550.3±81.1
PCM + GM (100 mg/kg)	259.8±10.8*	103.2±9.9*	455.1±86.1
One-way ANOVA F (df)	9.4(3,20)	12.0 (3,20)	1.3 (3,20)

Value are Mean±SEM; n= 6

@ *P*<0.05, When compared to control vs toxin control group

* *P*<0.05, when compared to toxin control

In the control group, histopathology of liver showed normal hepatic cells with well-preserved cytoplasm, nucleus, central vein and portal triad (fig. 1a). Liver section of rats in pre and post-treated toxin control groups of paracetamol showed the highest degree of damage i.e., ballooning degeneration, necrosis, degenerated nucleus, dilated sinusoids and inflammation (fig.1b). The liver section of rats post-treated with silymarin showed almost normal hepatic cells and sinusoids (fig. 1c). *Gymnosporia montana* (Roth) Bemth. extract (100 mg/kg) was more effective than low dose (50 mg/kg) and comparable to silymarin-treated group (figs. 1d and 1e). Post-treatment with *Gymnosporia montana* (Roth) Bemth. extract in the dose of 100 mg/kg showed almost normal architecture and lesser degree of ballooning degeneration as compared to toxin group. The nucleoli were prominent with occasional binucleate cells.

**Fig. 1:**
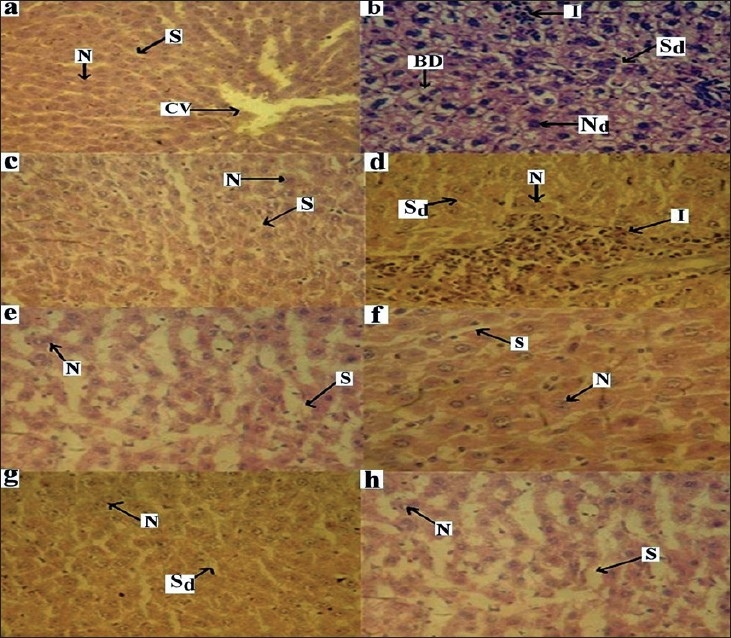
Effect of GM in PCM induced hepatotoxicity in rats. (a): Control group showing normal architecture, central vein (CV), hepatic cell (N) and Sinusoids (S). (b): Post and pre-treated toxin paracetamol control group: showing inflammation (I), ballooning degeneration (BD), degenerated hepatic cell (N_d_) and dilated sinusoids (S_d_). (c): Posttreated silymarin group: showing normal hepatic cell and sinusoids. (d): Post-treated GM (50 mg/kg) group: showing less inflammation (I) and dilated sinusoid (S_d_) and normal hepatic cell. (e): Post-treated GM (100 mg/kg) group showing normal hepatic cell and sinusoids. (f): Pre-treated silymarin group: showing normal hepatic cell and sinusoids. (g): Pre-treated GM (50 mg/kg) group: showing less dilated sinusoid (S_d_) and normal hepatic cell. (h): Pre-treated GM (100 mg/kg) group: showing normal hepatic cell and sinusoids, magnifi cation 40X; stain Haemotoxylin-eosin.

The liver section of rats pre-treated with silymarin showed normal hepatic cells and sinusoids (fig. 1f). In pre-treatment with 50 and 100 mg/kg of *Gymnosporia montana* (Roth) Bemth. extract (figs. 1g and 1h), little ballooning degeneration was seen in comparison to toxin group. Sinusoids and architecture of liver were almost normal as seen in control group. The recovery of liver section of rats pre-treated with *Gymnosporia montana* (Roth) Bemth. extract (100 mg/kg) were almost identical to that seen in silymarin-treated group.

The liver can be injured by many chemicals and drugs. Paracetamol is a known antipyretic and analgesic which produces hepatic necrosis in man, rats and mice with toxic doses. In the present study, paracetamol was selected as a hepatotoxin to induce liver damage. In therapeutic doses, paracetamol is metabolized by glucuronide and sulfate conjugation (major pathway). N-acetyl-p-benzoquinoneinoneimine (NAPQI) is a highly toxic minor metabolite formed by cytochrome P-450 (minor pathway). This little amount of toxic metabolite is detoxified by conjugation with glutathione. The glucuronide and sulfate conjugation pathway become saturated and large amount of toxic metabolite is formed in high doses, which cannot be detoxified through glutathione conjugation. This metabolite bind covalently to unsaturated lipid membrane lead to lipid peroxidation followed by enzymatic dysfunction, decreased protein synthesis, depletion of glutathione, triglyceride accumulation, necrosis and cell death. The elevated levels of serum marker enzymes are indicative of cellular leakage and loss of functional integrity of cellular membrane in liver. This is evident from an elevation in the levels of SGOT, SGPT and ALP (*p*<0.05) as compared to control group[16,17].

Post-treatment with *Gymnosporia montana* (Roth) Bemth. extract in the dose of 50 and 100 mg/kg reduced the paracetamol-induced elevations in SGOT (*p*<0.05) and SGPT (*p*<0.05) compared to toxin control. Reduction in the ALP (p<0.05) was seen in the *Gymnosporia montana* (Roth) Bemth. extract in the dose of 100 mg/kg compared to toxin control. The liver section of rats post-treated with *Gymnosporia montana* (Roth) Bemth. extract in the dose of 50 and 100 mg/kg were effective in paracetamol-induced hepatotoxicity. Post-treatment with *Gymnosporia montana* (Roth) Bemth. extracts (100 mg/kg) revealed hepatoprotective activity similar to the silymarin-treated group.

Pre-treatment with *Gymnosporia montana* (Roth) Bemth. extract in the dose of 50 and 100 mg/kg lowered the paracetamol-induced elevations in SGOT (*p*<0.05) and SGPT (*p*<0.05) compared to the toxin control group. Reduction in the ALP (*p*<0.05) was not significant in the *Gymnosporia montana* (Roth) Bemth. extract (50 mg/kg and 100 mg/kg) compared to control and toxin control group. Histopathological examination of the liver section of the rats treated with *Gymnosporia montana* (Roth) Bemth. extracts (50 and 100 mg/kg) exhibited less degree of damage as compared to toxin control group. Pre-treatment with *Gymnosporia montana* (Roth) Bemth. extracts (100 mg/kg) revealed better hepatoprotective activity similar to the silymarin-treated group.

The pre and post-treated groups treated with *Gymnosporia montana* (Roth) Bemth. extract in the dose of 100 mg/kg were comparable to the silymarin-treated group. Rats post-treated with *Gymnosporia montana* (Roth) Bemth. extract in the dose of 100 mg /kg exhibited significant lowering of the serum markers than pre-treatment group. *Gymnosporia montana* (Roth) Bemth. leaves have been reported to contain beta-amyrin, beta-sitosterol, and delta-amyrin[18,19]. The observed hepatoprotective effect of the *Gymnosporia montana* (Roth) Bemth. extract against paracetamol-induced hepatotoxicity may be attributed to the presence of beta-amyrin and beta-sitosterol[20–22]. Beta-amyrin is known to decrease the leakage of serum marker enzymes SGOT, SGPT, ALP, replenish the depleted hepatic GSH, reduce the liver damage and suppress the cytochrome P450 leading to hepatoprotection. These findings are indicative of the hepatoprotective potential of beta-amyrin against liver injury, oxidative stress and toxic metabolite formation. Many compounds, known to be beneficial against paracetamol-induced liver injury exert their protective action by either replenishing the depleted hepatic GSH or suppressing the cytochrome P450[23]. The ethanol extract of *Gymnosporia montana* (Roth) Bemth. exerted its protective action against paracetamol-induced hepatotoxicity by decreasing the elevated serum marker enzymes and possibly by replenishing the depleted hepatic GSH, suppressing the cytochrome P450 and thus preventing liver damage. The present study indicates that ethanol extract of *Gymnosporia montana* (Roth) Bemth. Possesses significant hepatoprotective activity.
